# Dietary patterns and breast cancer risk among women in northern Tanzania: a case–control study

**DOI:** 10.1007/s00394-012-0398-1

**Published:** 2012-06-23

**Authors:** Irmgard Jordan, Antje Hebestreit, Britta Swai, Michael B. Krawinkel

**Affiliations:** 1 Institute of Nutritional Sciences, Justus-Liebig-University Giessen, Wilhelmstr. 20, 35392 Giessen, Germany; 2 Bremen Institute for Prevention Research and Social Medicine, Bremen, Germany; 3 Kilimanjaro Christian Medical Center, Moshi, Tanzania

**Keywords:** Breast cancer, Dietary pattern, PUFA, Tanzania

## Abstract

**Background:**

Breast cancer is the second most common cancer among women in the Kilimanjaro Region of Tanzania. It was tested within a case–control study in this region whether a specific dietary pattern impacts on the breast cancer risk.

**Methods:**

A validated semi-quantitative Food Frequency Questionnaire was used to assess the dietary intake of 115 female breast cancer patients and 230 healthy age-matched women living in the same districts. A logistic regression was performed to estimate breast cancer risk. Dietary patterns were obtained using principal component analysis with Varimax rotation.

**Results:**

The adjusted logistic regression estimated an increased risk for a “Fatty Diet”, characterized by a higher consumption of milk, vegetable oils and fats, butter, lard and red meat (OR = 1.42, 95 % CI 1.08–1.87; *P* = 0.01), and for a “Fruity Diet”, characterized by a higher consumption of fish, mango, papaya, avocado and watery fruits (OR = 1.61, 95 % CI 1.14–2.28; *P* = 0.01). Both diets showed an inverse association with the ratio between polyunsaturated and saturated fatty acids (P/S ratio).

**Conclusion:**

A diet characterized by a low P/S ratio seems to be more important for the development of breast cancer than total fat intake.

## Introduction

Established factors for breast cancer development are age at menarche, age at menopause, age at first full-term pregnancy, breastfeeding and alcohol consumption at all ages [1–7]. A high percentage of total body fat and tall height at adulthood in postmenopausal women is associated with an increased risk [8–11]. Several studies have looked at possible linkages between single nutrient intake as well as foods or dietary patterns and breast cancer [12–18]. However, there has been limited evidence suggesting that consumption of total dietary fat and special dietary patterns influence breast cancer risk, but no internationally accepted conclusion was reached up to now [7, 19, 20].

In Tanzania, a low-income country where breast cancer is currently the second most common cancer in women, the lifestyle characterized by long-standing lactation or late age at menarche has been associated with a lower breast cancer risk [21]. However, breast cancer occurs, and a pilot case–control study in the Kilimanjaro Region of northern Tanzania estimated an increased association between alcohol consumption and breast cancer [22]. A new case–control study looked at dietary patterns rather than single nutrients as nutrients are ingested within diets. A case–control design was chosen because of a lack of demographic data and infrastructural deficits for identification of all women affected to allow for a prospective study approach.

The study was carried out in collaboration between the Kilimanjaro Christian Medical Centre (KCMC) in Moshi, Tanzania, and the Institute of Nutritional Sciences of the University of Giessen, Germany.

## Methods

Breast cancer patients and controls were recruited in the Kilimanjaro Region between 2004 and 2007. The detailed study methodology has been described previously [21]. In summary, cases were identified using fine needle aspiration cytology (FNAC) confirming primary breast cancer diagnosis. The hospital and visitor-based controls were matched according to age (±1.5 years) and lived in the same district for at least 5 years during the past 10 years. The controls were interviewed for their medical history and underwent a physical examination to exclude palpable breast cancer. After informed consent, 115 cases and 230 controls were interviewed by either a trained nurse or a medical doctor in Swahili, based on a standardized questionnaire in English about their socioeconomic situation, current and former lifestyle. At first, the variables were tested for normal distribution, followed by their respective tests for statistically significant differences between cases and controls. The two control groups were analysed for differences in their socioeconomic status using the Mann–Whitney *U* test [21].

The present analyses focus on the dietary patterns of both cases and controls using the data of a semi-quantitative food frequency questionnaire (FFQ). The FFQ food list was prepared based on market surveys at different seasons and completed after a pre-test. The relative validity of the FFQ was assessed in 2005 and 2006 based on two non-consecutive 24-h recalls of 50 randomly selected women with a mean age of 40 years (23–70 years), who did not participate in the case–control study but lived in the same study region. The validation study covered two seasons with different food availability: dry and rainy season. Data collection was done by four trained enumerators. The training included estimation of quantities using common household measurements, for example, cups, spoons, customary packing size, and solid foods in pieces or slices. Foods were prepared according to local standard recipes and weighed using household kitchen scales by the research staff. Countable foods such as onions, eggs or bananas were classified according to their size into small, medium and large. Samples of food pieces were obtained from the local market, and mean weights were taken of each size. The matter of size was intensively discussed in the interviewer trainings to assure a common comprehension. A raw/cooked coefficient was applied when large deviations between cooked and raw foods were expected after preparation, for example, for dried cereals (pasta, rice) and dried legumes. The coefficients were calculated by cooking experiments done by the nutritionist but without calculating any loss of vitamins and minerals. Seasonal food availability on individual level was assessed within the interview, especially for fruits, and a seasonal factor was applied accordingly.

The FFQ data from both, the validation and the case–control study, were entered into NutriSurvey^®^, a nutrition software package, which generated tables of the individual food and nutrient intake per day, latter based on food composition tables from Tanzania, Kenya, Senegal, Mali and Germany [23, 24]. All data were converted to gram intake per day for each food item.

For the validation study, the data sets were merged into six food groups to describe individual food intake: (1) cereals: bread, rolls, cereal products, grains, egg-free pasta; (2) vegetables: vegetables, pulses, potatoes, mushrooms; (3) animal products: eggs, dairy and cheese, meat, fish, poultry, sausages and other meat products; (4) beverages: non-alcoholic beverages, coffee, tea, water, alcoholic beverages; (5) fruits; (6) fats: oil, fats, butter. Since the values of most variables were not normally distributed, non-parametric tests were carried out in the subsequent analysis. The studied population had a low educational level, and considering the relative high number of interviewers in relation to the study population, the validation data were tested for interviewer effects before any statistical analysis was performed. The Kruskal–Wallis test chosen to test for homogeneity between the interviewers showed interviewer effects in 100 % of the food groups confirmed by the median one-way test at a level of 83 %. Therefore, further analysis was carried out stratified by interviewer. The Wilcoxon signed rank test was used to test the 24-h recall and the FFQ for seasonal variability. It is a non-parametric test equivalent to the paired *t* test. In addition, the Wilcoxon signed rank test was used to test for differences in the results of the 24-h recall and FFQ. There was no evidence for a seasonal effect in the food groups if the FFQ is used, except for non-alcoholic beverages. Differences in the intake of oils and fats assessed by the validated FFQ and its reference, the 24-h recall, could only be shown by one interviewer. This might be due to low quantification capacities of the studied population especially in this respective food group and especially during the 24-h recall. Furthermore, Spearman correlation was calculated with all interviewers grouped together for comparison with other studies that did not report whether they checked for interviewer bias. The correlation coefficient (*r*_s_) was highest in the food group “fruits” (*r*_s_ = 0.39, *P* = 0.01) followed by “cereals” (*r*_s_ = 0.38, *P* = 0.01), “beverages” (*r*_s_ = 0.33, *P* = 0.01) and the food groups “animal products” and “vegetables” (*r*_s_ = 0.27 and *r*_s_ = 0.14, respectively, both values not significant). A negative non-significant correlation coefficient (*r*_s_) was found in the food group “oils and fats” (*r*_s_ = −0.22, *P* = 0.13). There had been no consistent statistical differences between FFQs and the 24-h recalls, and the correlations were low to modest but comparable to other studies except for oil and fats [25–27]. The negative correlation coefficient for oils and fats might be explained by the difficulties in assessing the oil and fat consumption using the 24-h recall. The reference methods ranged from 7-day-weighed record and 2-day-weighed record to two 7-day food dairies. The high variation in correlation coefficients for the different food groups might be caused by under- or overestimation due to either high fluctuations in food availability or difficulties on the part of the respondents in estimating the quantities of the foods consumed in low-income countries. However, Parr et al. [28] pointed out that these factors should not be directly linked to the questionnaire design; thus, the tested FFQ was considered a reliable instrument to assess dietary intake in the Kilimanjaro Region.

However, it was recommended paying special attention to the training of interviewers and especially to the assessment of the oil and fat intake. In addition, a calculation for seasonal variability in fruit and vegetable intake was recommended to be used where applicable. The FFQ finally contained in total 65 food items.

From data on individual food intake of the case–control study population, dietary patterns were created using PCA. Although there is a certain disagreement among statistical theorists about it [29–31], PCA was chosen for keeping the results comparable to other studies looking at dietary patterns and disease [20, 32–36]. The sampling adequacy of the food group variables for factor analysis was confirmed using the Kaiser–Meyer–Olkin measure. The food items listed in the FFQ were at first merged into 36 food groups for obtaining factors from the PCA defined as dietary patterns. A second PCA was performed based on 34 food groups, excluding alcoholic beverages. Scree plots and parallel analysis were used to quantify the number of factors wanted [31]. Food groups with factor loadings between −0.4 and 0.4 were disregarded for defining the dietary patterns. Differences in body size, metabolic efficiency and physical activity increase the variation in dietary intake, thus requiring energy adjustment. We chose to apply the residual method after performing the PCA to ensure comparability between the dietary patterns [37, 38]. The final dietary patterns were included in a non-conditional logistic regression model; at first adjusted only for age. Secondly, the dietary patterns were included into the basic model described elsewhere [21]. This model includes the matching variables of age, place of living and the acknowledged predictors in the aetiology of breast cancer from high-income countries. In addition, the body mass index (BMI) of the women at the age of 10 and 20 years as well as current BMI was estimated by each woman herself using a pictogram developed by Stunkard et al. [39] and modified to African settings [21].

If possible, the variables were entered as continuous variables. The variable “age at first full-term pregnancy” was categorized into three groups, “first full-term pregnancy ‘≤20 years’, ‘>20 years’, and ‘no pregnancy’”.

Descriptive statistics, principal component analysis and logistic regression were performed using the statistical package of SPSS version 18 (SPSS Inc.).

Ethical clearance was obtained from the Research and Ethics Committee of the KCMC, Moshi, Tanzania, and the Ethics Committee of the Faculty of Medicine of the University of Giessen, Germany.

## Results

Selected socioeconomic and reproductive characteristics of the study participants are presented in Table 1 [21]. Mean age of all women was 50 years, and 94 % of them had children. Mean age at menarche was 16 and 21 years at delivery of the first child. Mean lifelong lactation time was 88 months. Breast cancer patients had a significantly lower lifelong lactation time compared to controls. The basic logistic model estimated an increased risk for women with a higher BMI at 20 years, but a reduced risk for women with a high property level and prolonged lactation (OR_BMI 20 years_ = 1.31; 95 % CI, 1.11–1.55; OR_high property_ = 0.22; 95 % CI, 0.09–0.55; and OR_lactation_ = 0.99; 95 %CI, 0.98–1.00; all *P*s < 0.01).

**Table 1:** Selected socioeconomic and reproductive indicators [21]

Variable	Cases			Controls			*P* value*
	Median (min–max)		*n*	Median (min–max)		*n*	
Age (years)	50 (28–85)		115	50 (26–83)		230	0.620
Age at menarche (years)	16 (11–20)		111	16 (13–20		230	0.267
Age at first full-term pregnancy (years)	20 (14–35)		106	20 (13–41)		217	0.571
Number of children^a^	5 (1–10)		106	5 (1–9)		217	0.219
Lifelong lactation (months)	90 (0–240)		114	108 (0–240)		230	0.045
Schooling (%)							0.119
Less than 3 years	27			18			
Finished primary school	54			59			
Finished secondary school	19			23			
Property level (%)							<0.0001
Low	47			18			
Medium	45			65			
High	8			17			
Women with children (%)	92			94			0.515

* Mann–Whitney *U* test: differences between cases and controls

^a^Only parous women

Median energy consumption in all women was 1,714 kcal per day (min 786 kcal; max 3,928 kcal), median protein intake was 47 g/day (min 17 g/day; max 183 g/day), median fat intake was 72 g/day (min 30 g/day; max 166 g/day) and median carbohydrate intake was 188 g/day (min 85 g/day–max 537 g/day). Median percentage of food energy from protein was 12 %, from fat 39 % and from carbohydrates 46 %. Median alcohol intake from alcoholic drinks was 8.2 g/day (min, 0 g/day; max, 100 g/day). Main alcoholic drinks were *Mbege* (often homemade, locally brewed beer), bottled beer and wine (median intake 57 g/day, min 0 g/day; max 298 g/day and 0 g/day, min 0 g/day; max 77 g/day respectively).

**Table 2:** Results of rotated principal component analysis (PCA 1)

Food item	Diet of the Rich	*Mchicha* Diet	Banana Diet	Fatty Diet
Variance explained (%)	**9.1**	**7.9**	**7.6**	**5.3**
Rice	0.618	0.205	−0.143	−0.170
Nuts	0.587	−0.006	0.124	−0.089
Egg	0.557	−0.039	0.162	0.043
*Chapati* ^*a*^	0.556	0.062	0.055	0.009
Leguminous vegetables	0.537	−0.093	0.006	−0.026
Bread	0.503	0.362	−0.220	−0.190
Soda drinks	0.471	0.108	−0.028	−0.155
Red meat	0.453	0.103	−0.037	0.367
*Mchicha* ^b^	−0.017	0.645	0.029	0.110
Cucumber and okra	0.209	0.581	0.032	0.038
Onion	0.089	0.579	−0.042	0.138
Carrots and tomatoes	0.145	0.516	−0.096	−0.007
Maize	−0.180	0.461	0.135	−0.085
Fish	−0.018	0.434	0.337	−0.085
Avocado	−0.016	0.413	0.347	0.067
Banana	0.145	0.030	0.667	0.073
Green (cooking) banana	0.086	0.008	0.616	−0.176
Sugar	0.153	−0.103	0.491	−0.166
Watery fruits^c^	0.085	0.189	0.478	−0.218
Starchy tubers	−0.275	−0.063	0.461	0.136
*Mbege* ^d^	−0.295	0.050	0.442	0.246
Pulses	−0.070	0.281	0.415	0.134
Sunflower oil	0.203	−0.207	−0.071	−0.623
Milk	0.264	−0.079	−0.042	0.521
Butter and lard	−0.213	−0.254	0.055	0.457
Mixed vegetable fats and oil	0.263	0.191	−0.115	0.454
Tea	0.055	0.013	0.366	−0.410

Food groups with factor loadings <0.4 and >−0.4: potatoes, juice, chicken meat, mango, papaya, cabbage (white), *mandazi* (East African donuts), *uji* (thin millet or maize-based porridge), coffee, bottled beer and wine. Rotation method Varimax with Kaiser normalization. Rotation converged in 7 iterations

^a^Unleavened East African flat wheat bread, ^b^ traditional Tanzanian food, synonymously used for a dish of amaranth leaves and, for example, onions, tomatoes and/or carrots in various amounts, ^c^ oranges, watermelon and pineapple, ^d^ often home-made opaque beer from bananas and millet

A PCA was conducted primarily on 36 food groups with Varimax rotation. The Kaiser–Meyer–Olkin measure verified the sampling adequacy for the PCA, KMO = 0.621, which is considered as mediocre [40, 41]. Following Kaiser’s criterion retaining all components with eigenvalues greater than one, 14 components would have been useful for further analysis. However, the number of food groups with factor loadings <−0.4 or >0.4 varied between 0 and 11; thus, the results were not interpretable. Consequently, it was decided to retain four components as suggested by the scree plot. These four components or dietary patterns describe 29.9 % of the variance in food intake (Table 2). The first pattern is characterized by rice, nuts, eggs, *chapati* (unleavened East African flat wheat bread), leguminous vegetables, bread, soda and red meat. Since most of these food items are usually purchased, we called it the “Diet of the Rich”. Pattern two is characterized by *Mchicha*, cucumber, okra, onions, carrots, tomatoes, maize, fish and avocado. *Mchicha* is the Swahili name for amaranth leaf, a traditional food in Tanzania often synonymously used for a dish consisting of amaranth leaves and, for example, onions, tomatoes and/or carrots in various amounts. The pattern was therefore named “*Mchicha* Diet”. The third pattern is characterized by ripe and green banana, sugar, different fruits, tubers, pulses and *Mbege*. The mountainous area of the Kilimanjaro Region is known for its various banana plants. Therefore, pattern three was called “Banana Diet”. Pattern four is characterized by a high consumption of milk, butter, lard, vegetable oils and fats, and a low consumption of sunflower oil and tea. All of the positively loading food items relate to fat, thus we called this pattern “Fatty Diet”. With increased affiliation to this Fatty Diet, bread consumption decreased (1st quartile median = 17 g bread/d, 4th quartile median = 9 g bread/d; *P* for trend < 0.001) and red meat consumption increased (1st quartile median 44 g/day, 4th quartile median = 52 g/day; *P* for trend = 0.09).

**Fig. 1:**
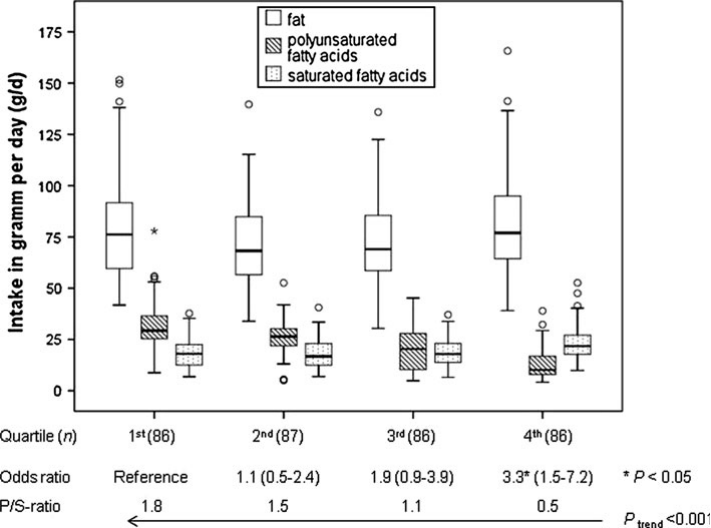
Intake of fat, polyunsaturated and saturated fatty acids per day and its related odds and P/S ratios in quartiles of the Fatty Diet

The non-conditional multivariate and logistic regression examining the associations between dietary behaviour and breast cancer showed an increased risk association with three out of the four dietary patterns: the *Mchicha*, Banana and Fatty Diets (Table 3). After including socioeconomic parameters and reproductive variables in the logistic model, the odds ratio (OR) for the *Mchicha* Diet changed from a significant OR of 1.47 (95 % CI, 1.14–1.88; *P* < 0.01) to a non-significant OR of 1.28 (95 % CI, 0.97–1.7; *P* = 0.08). The Banana and the Fatty Diets were still associated with an increased breast cancer risk on a significant level. The OR for the Fatty Diet increased to 3.04 (95 % CI: 1.34–6.91; *P* < 0.01) among women with the highest consumption (4th quartile). With increased affiliation to the Fatty Diet, total fat intake increased significantly (*P* = 0.04), whereas percentage of energy from fat did not change (*P* = 0.83) and whereas the ratio of polyunsaturated fatty acids to saturated fatty acids (P/S ratio) was inversely associated with breast cancer risk (Fig. 1). However, there was no risk association found between total fat intake (median 72 g/day) and breast cancer. In addition, there was no change found in risk associations if energy was included into the risk model described above (OR energy = 1.00, 95 % CI, 1.00–1.00; *P* = 0.51).

The Banana Diet includes *Mbege*—a local, often home-made opaque beer from bananas and millet. Acknowledging that alcohol is an accepted risk factor for breast cancer, the factor analysis was repeated excluding the alcoholic beverages from the food group list. In order to get comparable results to the first PCA, we generated six dietary patterns that described 40.3 % of the dietary variance, and four of them were comparable to the Diet of the Rich, *Mchicha*, Banana and Fatty Diets of the first PCA. Table 4 presents the results of the logistic regression including the second set of dietary patterns with the alcoholic beverages included as separated variable. The *Mchicha* Diet and the Banana Diet were no longer associated with breast cancer risk, but the new Fruity Diet and again a Fatty Diet very similar to the first Fatty Diet were associated with increased risk (OR 1.61, 95 % CI, 1.14–2.28; *P* = 0.,01 and OR 1.42, 95 % CI 1.08–1.87, *P* = 0.01, respectively). After energy adjustment, the OR for the Fatty Diet declined to 1.43 (95 % CI, 1.04–1.98; *P* = 0.014), whereas the OR for the Fruity Diet remained at the same level (OR 1.43, 95 % CI, 1.04–1.98; *P* = 0.03).

**Table 3:** Results of the logistic regression: dietary patterns only

Variable	*P* value	Odds ratio	95 % CI	*n*
Dietary patterns (PCA 1)				
Diet of the Rich	0.95	1.01	0.79–1.30	345
*Mchicha* Diet	0.00	1.47	1.14–1.88	345
Banana Diet	0.00	1.94	1.43–2.63	345
Fatty Diet	0.00	1.62	1.26–2.07	345

Adjusted for age

Constant: *P* value < 0.01; OR, 0.56; Cox and Snell *R*² = 0.13; Nagerkerke *R*² = 0.18

Overall percentage correctly classified, 74 %

**Table 4:** Basic breast cancer risk model and dietary patterns

Variable	*P* value	Odds ratio	95 % CI	*n*
Property level				
Low				87
Medium	0.00	0.37	0.20–0.71	198
High	0.01	0.27	0.09–0.77	48
Body mass index (kg/m²)				
At 20 years	0.01	1.27	1.06–1.53	333
At interview	0.09	0.93	0.85–1.01	333
Age at first full-term pregnancy				
≤20 years				193
>20 years	0.06	1.83	0.97–3.45	122
No pregnancy	0.80	0.82	0.18–3.84	18
Lifelong lactation	0.02	0.99	0.98–1.00	333
Dietary patterns (no alc)				
Diet of the Rich (no alc)	0.17	1.28	0.90–1.59	333
Energy adjusted*	*0.54*	*1.13*	*0.77*–*1.66*	
Fruity Diet (no alc)	0.01	1.61	1.14–2.28	333
Energy adjusted*	*0.03*	*1.43*	*1.04*–*1.98*	
* Mchicha* Diet (no alc)	0.70	1.06	0.80–1.40	333
Energy adjusted*	*0.70*	*1.06*	*0.80*–*1.40*	
Banana Diet (no alc)	0.12	1.32	0.93–1.87	333
Energy adjusted*	*0.30*	*1.21*	*0.84*–*1.75*	
Starchy Diet (no alc)	0.86	1.02	0.78–1.34	333
Energy adjusted*	*0.93*	*1.02*	*0.72*–*1.43*	
Fatty Diet (no alc)	0.01	1.42	1.08–1.87	333
Energy adjusted*	*0.01*	*1.43*	*1.08*–*1.90*	

Adjusted for age, place of living, age at menarche, menopausal status, *Mbege* (often home-made opaque beer*)*, beer and wine

Constant *P* value = 0.64; OR, 0.30; Cox and Snell *R*² = 0.21; Nagelkerke *R*² = 0.29; overall percentage correctly classified = 77 %

* Residual method after PCA

## Discussion

Several dietary patterns from two principal component analyses with Varimax rotation based on a FFQ were associated with increased breast cancer risk. Two patterns, both called Fatty Diet, are basically characterized by a higher consumption of milk, mixed vegetable oils and fats, butter and lard, but a low consumption of sunflower oil. Both Fatty Diets were associated with an increased risk in different logistical models. A diet rich in fat similar to our Fatty Diets was discussed by Schulz et al. [42] using reduced rank regression, stating that specific fatty acids are less important in populations with a generally higher fat consumption (mean 8.3–10.4 g/MJ). However, this level of dietary fat intake as a proportion of energy intake was comparable to our study population (mean 10.2 g/MJ), but the mean total fat intake in our population was 15 g per day lower because of the overall lower energy consumption than reported by Schulz et al. [42]. Here, the women’s total fat intake was not associated with breast cancer risk (data not shown), although total fat intake increased significantly with increased affiliation to the fatty dietary patterns. Another prospective cohort study found a direct association between dietary fat intake including subtypes and post-menopausal invasive breast cancer [43]. However, they recorded at median 20.3 % energy intake from fat per day in the 1st quintile and 40.1 % energy intake from fat per day in the last quintile. Only the latter energy intake level from dietary fat is comparable to our data. The wide range of fat intake observed in their study population may have resulted in an increased statistical power. This assumption was made by Thiébaut et al. [43] based on the hypotheses from Wynder et al. [44] that a threshold effect may exist for dietary fat, such that it would be difficult to detect an association between fat intake and breast cancer risk in Western populations. They referred to studies about Asian diets in which more people consume diets containing 20 % or less of energy from fat, which have shown significant or borderline significant associations of fat intake and breast cancer risk [43]. The median fat intake as percentage from energy intake in our study population was 39 %, which is above this benchmark of 20 % and may explain why no association was found for our population. Regarding the fatty acid composition of the diet, the major PUFA sources reported by Thiébaut et al. were vegetable oils and fats, butter and mayonnaise [43]. Except mayonnaise, these food items have also been identified by Schulz et al. [42] and in our study as part of dietary patterns rich in fat which have been associated with a higher risk of developing breast cancer. Even if the fatty acid composition of foods varies intrinsically, this composition may be more important than the total fat intake. Our study population showed a negative association of the P/S ratio with breast cancer risk (Fig. 1). This negative association was also observed in a case–control study among pre-menopausal women in Singapore, but was attributed to PUFA intake only [12]. However, results from the European Investigation into Cancer and Nutrition (EPIC) study [45] and a case–control study in Connecticut [46] supported the hypothesis of Rose et al. [47] and Key et al. [9] that both saturated and polyunsaturated fatty acids influence inversely the oestrogen metabolism and mammary carcinogenesis. In addition, results from the Shanghai Women’s Health study, a prospective cohort study, suggested that the relative amounts of n−6 PUFA to marine-derived n-3 PUFAs may be more important for the breast cancer risk than individual amounts of these fatty acids in the diet [48]. They supported the hypothesis that the different PUFA compete as enzyme substrates inside membrane phospholipids [49]: this may also explain the contradictory results of other studies analysing the effect of PUFAs on breast cancer risk [50–52].

Investigators from the Black Women’s Health Study, a prospective cohort study, identified a dietary pattern similar to our Fatty Diet called “Western Diet” also based on a PCA with Varimax rotation and factor loadings for dairy products and meat at similar level [53]. However, they associated a lower risk for breast cancer only with another dietary pattern, the “Prudent Diet”, characterized with a low consumption of meat and dairy products. Since both the Western and the Prudent Diets were more complex than in our study with each diet having more than 8 foods with factor loadings above 0.4, it is not known whether the non-relationship between the Western Diet and breast cancer has been masked by a higher consumption of potentially preventive foods which in turn result in a high P/S ratio.

### Effect of alcohol on dietary patterns risk association

The reported consumption of the local banana beer, *Mbege*, increased significantly with increased affiliation to the Fatty Diet (*P* for trend < 0.001). Our data show a higher breast cancer risk for women mainly following the Banana Diet, which was also associated with a high consumption of *Mbege*, even though there is no risk association between alcohol intake and breast cancer risk in this study. According to the WRCF panel, there is ample and generally consistent evidence from case–control and cohort studies that alcoholic drinks are a cause of pre- and post-menopausal breast cancer [7]. In order to exclude a possible bias in the risk estimation of dietary behaviour and breast cancer risk, we generated a second set of dietary patterns excluding the alcoholic beverages from the factor analysis. The risk-increasing effect of the Fatty Diet remained slightly less pronounced when alcoholic beverages were singularized and added separately into the risk estimation model. On the contrary, the *Mchicha* Diet and the Banana Diet were no longer associated with breast cancer risk, although the latter is characterized by rapidly absorbable carbohydrates. Such carbohydrates have been associated with increased breast cancer risk [20, 54]. One would assume that if the alcoholic beverages did influence the risk estimation of the *Michicha* and Banana Diets, the analysis keeping alcoholic beverages as separate food groups should visualize an increased risk association. However, the odds ratio of *Mbege* as well as bottled beer and wine was estimated to be 1.00 (95 % CI, 1.00–1.00; *P* = 0.08 and 0.87, respectively) indicating no risk association. In our study, the alcohol consumption was 8.2 g/day, which is well below the recommended maximum intake of one drink per day in the European code against cancer [55]. Thus, the alcohol intake in general was probably too low to show an effect.

### Fruity Diet

**Fig. 2:**
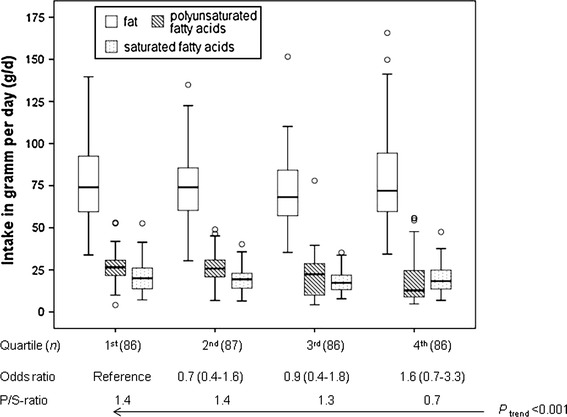
Intake of fat, polyunsaturated and saturated fatty acids per day and its related odds and P/S ratios in quartiles of the Fruity Diet

The Fruity Diet identified in the second PCA—keeping alcoholic beverages separate—was also associated with increased breast cancer risk. This diet is characterized by a high consumption of fish, mango, papaya, avocados and watery fruits like oranges, watermelons and pineapples that are known for their high content of valuable fatty acids, vitamins and micronutrients considered as potentially protective against cancer [56–60]. Nevertheless, several other studies could not show an overall association between fruit and vegetable intake and breast cancer risk [61, 62]. In our study, the Fruity Diet is, like the Fatty Diet, inversely associated with the P/S ratio (*P*_trend_ < 0.001), which is caused by a reduced intake of polyunsaturated fatty acids mainly from sunflower oil (*P*_trend_ < 0.001), but a stable saturated fatty acid intake (*P*_trend_ = 0.19). Thus, we concluded that it is not the fish and fruit intake but the accompanying dietary fat consumption that is associated with breast cancer (Fig. 2).

### Property level

Socioeconomic status (SES) is an internationally acknowledged indicator in epidemiological, economical and sociological studies. However, there is no international consensus on assessing SES, income and household expenditures being the most commonly used measures of SES [63]. In low-income countries like Tanzania, these indicators are difficult to assess. Often, poverty or possession scores are used instead. Studies from low-income countries looking at socioeconomic status and health have shown that a possession score might be even a better indicator of SES, as this score allows greater discrimination in identifying health risks than a poverty index [63]. In this study, a possession score called property, which was used as proxy for SES, showed an inverse association with breast cancer risk. This seems at odds with the statement that higher education and socioeconomic status are associated with an increased risk resulting from the lower number of parities and lactations. However, parity and lactation were correlated with educational level only. Furthermore, educational level was negatively correlated with age indicating a trend towards higher education among the younger women. Education might have an impact on breast cancer risk estimation in the future following the expectation that lactation and parity will reduce over time with increasing educational level. In addition, it is expected that the trend towards higher education and fewer children, thus reduced lifelong lactation, will continue especially with all the efforts towards the MDGs.

We did not find a correlation between lactation, parity and property level. In the context of this study, “low property level” means people can call a bicycle or a radio their own. If they own both, they already belong to the group at “medium property level”. Thus, any extra income is used first to improve basic living conditions like nutrition, sanitation and health before it is used for education. Alderman [64] points out that the relationship between possessions to nutrition provides only an indirect answer looking at social transfer programmes in low-income countries aiming at improvement in nutrition and healthcare-seeking behaviour. However, according to Hou et al. [65], it may not be surprising to observe an inverse association between SES and breast cancer risk, as studies have shown that people with low SES develop triple-negative subtypes, which accounts for a substantial proportion of breast cancer in Africa. Nevertheless, they required confirmation by larger population-based studies.

### Strengths and limitations

Our data show the impact of reproductive and lifestyle factors on breast cancer aetiology of women in the Kilimanjaro Region. With regard to eating habits and dietary patterns, the diversity of the Kilimanjaro diet is low, and it was less likely to miss important foods on the FFQ food list reducing the estimation bias for dietary behaviour. Due to low education levels and the poor infrastructure, we do not expect socially desirable answers, and participants are less likely to be informed about possible dietary impacts on health outcomes. The semi-quantitative FFQ allowed us to identify non-consumers and frequent consumers on the basis of eating habits, nutrient and energy intake in the case and control groups. Also, ready-to-use meals and eating out are uncommon in the Kilimanjaro region, which facilitated the identification of food groups based on single food items and less on complex meals.

The sample is relatively small compared to studies in Westernized countries. The sample size required to achieve a high level of power in a logistic regression depends on the number of predictors and the size of the expected effect. Peduzzi et al. [66] showed that no problems occur by events per variable (EVP) of 10 or more. Also, high regression coefficients and high correlations between the predictors may cause large problems in the estimation process, resulting in very low power even with EVP of 20 or more [67]. Thus, we have tested multicollinearity, which was acceptable in all predictors used. Several studies showed that a sample size like in our studies allows detecting large and medium effects, but might miss small effects. Thus, the results of our study are moderately powered and need to be confirmed by studies with a larger study population.

Before running a PCA, the sampling adequacy was controlled using the KMO measure. The KMO measure, which was 0.62, is considered as mediocre in our case [68, 69]. A low KMO measure might result in a high unexplained variance. However, in this study, we extracted 6 factors explaining 40.3 % variance, which is a medium result compared to other studies, for example: Hu et al. = 2 factors: 20 %; Arkkola et al. = 7 factors: 29.5 %; Shi et al. = 4 factors: 28.5 %; Lau et al. = 2 factors: 17.1 % [69–73]. In addition, Bartlett’s test for sphericity was 2,599.25, *P* < 0.001 indicating that correlations between items were sufficiently large for a PCA.

A major limitation is the PCA method. There have been discussions that the PCA method is less suitable for risk estimations of dietary patterns, because of difficulties to find plausible linkages between dietary patterns and the observed disease [30]. Therefore, it was recommend to use reduced rank regression based on response variables. However, breast cancer develops over a long period of time. Thus, using response variables—such as biochemical parameters—is only possible in prospective studies.

The knowledge about breast cancer and breast self-examination was very poor in our study population, and facilities for cancer diagnosis and treatment are still rare in countries like Tanzania [74]. In order to avoid a bias, we excluded the family history data for cancer from the analysis.

In the absence of a general health insurance, patients have had to pay for getting access to the health facilities. With the aim to minimize confounding errors due to different livelihood systems between cases and controls, we decided to select the controls also from within the hospital setting. But thereby, other selection biases cannot be excluded.

In conclusion, a dietary pattern rich in fat and characterized by a low P/S ratio may be associated with a higher risk of breast cancer. The fatty acid composition is probably more important than total fat intake for the breast cancer risk.
